# Specific Matrix Metalloproteinases Play Different Roles in Intraplaque Angiogenesis and Plaque Instability in Rabbits

**DOI:** 10.1371/journal.pone.0107851

**Published:** 2014-09-18

**Authors:** Xiao Qiong Liu, Yang Mao, Bo Wang, Xiao Ting Lu, Wen Wu Bai, Yuan Yuan Sun, Yan Liu, Hong Mei Liu, Lei Zhang, Yu Xia Zhao, Yun Zhang

**Affiliations:** 1 Key Laboratory of Cardiovascular Remodeling and Function Research, Shandong University Qilu Hospital, Jinan, Shandong, China; 2 Department of Traditional Chinese Medicine, Shandong University Qilu Hospital, Jinan, Shandong, China; 3 Department of Endodontics, Jinan Stomatologic Hospital, Jinan, Shandong, China; Baker IDI Heart and Diabetes Institute, Australia

## Abstract

**Background:**

Ectopic angiogenesis within the intima and media is considered to be a hallmark of advanced vulnerable atherosclerotic lesions. Some studies have shown that specific matrix metalloproteinases (MMPs) might play different roles in angiogenesis. Therefore, we investigated the predominant effects of specific MMPs in intraplaque angiogenesis and plaque instability in a rabbit model of atherosclerosis.

**Methods and Results:**

New Zealand rabbits underwent balloon injury of the abdominal artery and ingestion of a high-cholesterol (1%) diet to establish an atherosclerotic animal model. At weeks 4, 6, 8, 10, and 12 after balloon injury, five rabbits were euthanized and the abdominal aorta was harvested. Blood lipid analysis, intravascular ultrasound imaging, pathologic and immunohistochemical expression studies, and western blotting were performed. From weeks 4 to 12, the expression of MMP-1, -2, -3, and -9 and vascular endothelial growth factor A (VEGF-A) increased with atherosclerotic plaque development in the abdominal aorta, while the expression of MMP-14 substantially decreased. The vulnerability index (VI) gradually increased over time. Intraplaque neovessels appeared at week 8. The microvessel density (MVD) was greater at week 12 than at week 8. The VI, MVD, and VEGF-A level were positively correlated with the MMP-1, -2,-3, and -9 levels within plaques. Negative correlations were noted between the MMP-14 level and the VI, MVD, and VEGF-A level.

**Conclusion:**

Upregulation of MMP-1, -2, -3, and -9 and downregulation of MMP-14 may contribute to intraplaque angiogenesis and plaque instability at the advanced stage of atherosclerosis in rabbits.

## Introduction

Atherosclerotic plaque rupture is a major cause of acute cardiovascular events. Thus, stabilization of vulnerable plaques is of great clinical importance [1]. Pathological studies have identified specific characteristics of atherosclerotic plaques that are associated with plaque instability and rupture, including the ongoing inflammatory response, matrix degradation, and cell death. These changes result in eventual thinning of the fibrous cap and an increase in the inflammatory and necrotic core content. Neovascularization is another crucial feature of atherosclerotic plaques. The number of neovessels increases with plaque progression, and such vessels are abundant in vulnerable plaques [2]. Neovessels within plaques are characterized by fragility and high perfusion, thus allowing for extravasation of lipoproteins and red blood cells that contribute to the formation of plaque lipids [3]. This process results in intraplaque hemorrhage, increases the permeability of inflammatory cells, and leads to plaque destabilization [4], [5]. Ectopic angiogenesis within the intima and media is considered to be a hallmark of advanced vulnerable atherosclerotic lesions.

Angiogenesis is induced by various growth-inducing and -inhibiting factors. Multiple complex signal transduction pathways are involved in intraplaque angiogenesis. Proteinases are required for degradation of the extracellular matrix (ECM), creating an avenue for migrating endothelial cells during angiogenesis. The specific MMPs necessary for endothelial cell migration and tube formation [6] have attracted particular attention because they directly degrade ECM components. MMPs, also termed matrixins, are a family of more than 20 zinc-containing endopeptidases that degrade various components of the ECM [7]. MMPs are subdivided into at least five groups based on their structure and/or substrate specificities. MMP family members include collagenases (MMP-1, -8, -13, and -18), gelatinases (MMP-2 and -9), stromelysins (MMP-3, -10, and -11), matrilysins (MMP-7 and -26), and membrane-type MMPs (MMP-14 and -15).

It has become clear that MMPs contribute more to angiogenesis than just degrading ECM components. Various MMPs, including MMP-1, -2, -3, -9, and -14, have been shown to enhance angiogenesis [8]–[12]. Specific MMPs can also negatively contribute to angiogenesis [13]–[15]. However, the predominant effects of MMPs in intraplaque angiogenesis at the advanced stages of atherosclerosis remain inconclusive. In the present study, we investigated the roles of different MMPs in angiogenesis in patients with atherosclerosis.

## Materials and Methods

### Ethics statement

The experiment complied with the Animal Management Rule of the Ministry of Public Health, People’s Republic of China (documentation 55, 2001), and the experimental protocol was approved by the Animal Care Committee of Shandong University. All surgical procedures were performed with the rabbits under general anesthesia, and all efforts were made to minimize suffering.

### Animal protocol

Adult male New Zealand White rabbits (n = 52) weighing 1.7 to 2.1 kg were obtained from Jinan Xilingjiao Culture and Breeding Center (Jinan, Shandong Province, China). The animals were housed in individual cages at the Animal Care Center of Shandong University Qilu Hospital. All procedures were performed after general anesthesia had been induced. Maintenance of a slight corneal reflex was tested using saline drops.

A rabbit model of atherosclerosis was established as previously described [16]–[18] with modifications. All animals were fed an atherogenic diet (120–140 g/day of a 1% cholesterol and 99% standard rabbit diet) for 12 weeks. Twenty-seven rabbits underwent balloon-induced abdominal aortic endothelial injury under general anesthesia, while the other 25 rabbits underwent no injury (control group).Ten randomly chosen rabbits (aortic injury group, n = 5; control group, n = 5) were euthanized at the end of weeks 4, 6, 8, 10, and 12, and their abdominal aortas were harvested. Before euthanasia, the rabbits underwent intravascular ultrasound (IVUS) imaging to examine the morphological changes of the aortic plaques, and blood was drawn from the auricular artery after an overnight fast. The body weights of all rabbits were monitored throughout the experiment.

### Blood lipid analysis

Blood samples were centrifuged at 3000 revolutions per minute for 10 min at 4°C. Serum samples were then collected and stored at −80°C. The serum levels of total cholesterol (TC), high-density lipoprotein cholesterol (HDL-C), low-density lipoprotein cholesterol (LDL-C), and triglycerides (TG) were measured by enzymatic assays using an automated biochemical analyzer (Roche Hitachi 917; Block Scientific, NY, USA).

### IVUS assay

Each IVUS study was performed according to a standard procedure [19]. IVUS imaging was performed using a 3.2-F catheter containing a 40-MHz single-rotating-element transducer connected to an IVUS system (Galaxy; Boston Scientific, Fremont, CA, USA). The catheter was withdrawn to the abdominal aorta by a motorized pullback device at a constant speed of 0.5 mm/s. The lumen area (LA) and external elastic membrane area (EEMA) were measured on abdominal aortic cross-sectional images. The plaque area (PA) was calculated by EEMA – LA, and the plaque burden (PB, %) was calculated by PA/EEMA×100% [20].

### Histopathology and immunohistochemistry

The abdominal aorta (2 cm long) was fixed in 4% formaldehyde for 24 h, and 5-µm-thick segments were then serially sectioned. Frozen sections were stained with Oil Red O (Santa Cruz Biotechnology, Santa Cruz, CA, USA) to determine the lipid content, and paraffin sections underwent Sirius red, hematoxylin and eosin, and immunohistochemical staining.

Immunohistochemical staining was performed using standard techniques as previously described [21]. Briefly, endogenous peroxidase activity was inhibited by incubation with 3% hydrogen peroxide. Sections were blocked with 5% goat serum in phosphate buffered saline and incubated overnight at 4°C with primary antibodies. After washing with phosphate buffered saline, the sections were incubated with secondary antibody at 37°C for 30 min. The immunohistochemical staining results were analyzed using a diaminobenzidine kit (Zhongshan Goldenbridge Biotechnology, Beijing, China). Hematoxylin was used to counterstain the nucleus. The primary antibodies were mouse anti-rabbit RAM-11 (1∶200; Dako Glostrup, Denmark); α-smooth muscle cell (SMC) actin (1∶200; Sigma Chemical, Santa Clara, CA, USA); CD31, MMP-1, -2, -9, and -14, vascular endothelial growth factor A (VEGF-A), and collagen I (1∶20, 1∶100, 1∶100, 1∶50, 1∶100, 1∶100, 1∶100, respectively; Abcam, Cambridge, MA, USA); MMP-3 (1∶100; Chemicon, Boston, MA, USA); and collagen III (1∶200; Novus Biologicals, Littleton, CO, USA). The cross-reactivity between antibodies and rabbit antigens was tested in preliminary experiments and confirmed by negative-control experiments involving nonimmune IgG instead of primary antibodies.

Histopathological slides were analyzed using Image-Pro Plus 6.0 (Media Cybernetics, Cambridge, MA, USA). The intima–media thickness (IMT) of each aortic plaque was measured as follows. Eight randomly chosen fields in each cross section and five cross sections in each rabbit were selected for quantitative measurement, and the values were averaged [22]. The area of positive immunohistochemical staining was expressed as the proportion of the stained area divided by the total plaque area in at least 10 high-power fields (200×). The vulnerability index was calculated as follows: (macrophage staining % + lipid staining %)/(smooth muscle cell % + collagen fiber %) [21]. Ten random high-power fields (200×) were used for each sample to quantify the MVD in sections stained for CD31, and the microvessels were then quantified by the plaque area.

### Western blot analysis

Protein was extracted and separated on 10% to 15% SDS-PAGE gel and transferred onto nitrocellulose membranes. After blocking with 5% nonfat milk for 2 h at room temperature, the membranes were incubated with the following primary antibodies overnight at 4°C: anti-MMP-1 (1∶1000; Abcam, Cambridge, MA, USA), anti-MMP-2 (1∶1000; Abcam, Cambridge, MA, USA), anti-MMP-3 (1∶1000; Chemicon, Boston, MA, USA), anti-MMP-9 (1∶1000; Abcam, Cambridge, MA, USA), and anti-MMP-14 (1∶100; Abcam, Cambridge, MA, USA). After being washed in TBS-T, the membranes were incubated with horseradish peroxidase-conjugated secondary antibody for 2 h at room temperature. Signals were detected using an enhanced chemiluminescence kit (Millipore, Billerica, MA, USA). The protein levels were normalized to β-actin.

### Statistical analysis

All data analyses were performed using Predictive Analysis Software 18.0 (SPSS Inc., Chicago, IL, USA). Intergroup comparisons involved one-way ANOVA followed by the least-squares difference test (with equal variances assumed) or Dunnett’s T3 test (equal variances not assumed). Spearman’s rank correlation coefficient was used for correlation analysis. All data are presented as mean ± standard error of the mean. A two-tailed *P* value of <0.05 was considered statistically significant.

## Results

All rabbits showed full recovery without complications after balloon injury. Administration of the atherogenic diet was well tolerated by all rabbits, and no adverse effects were observed. Two rabbits that underwent balloon injury died of diarrhea at weeks 6 and 10.

In the control group, 12-week administration of the atherogenic diet resulted in fatty streak formation with lipid infiltration and only scarce plaques in the abdominal aorta (see supporting information for details; Figures S1–S4).

### Serum lipid assay and body weight of balloon-injured rabbits

**Figure 1:**
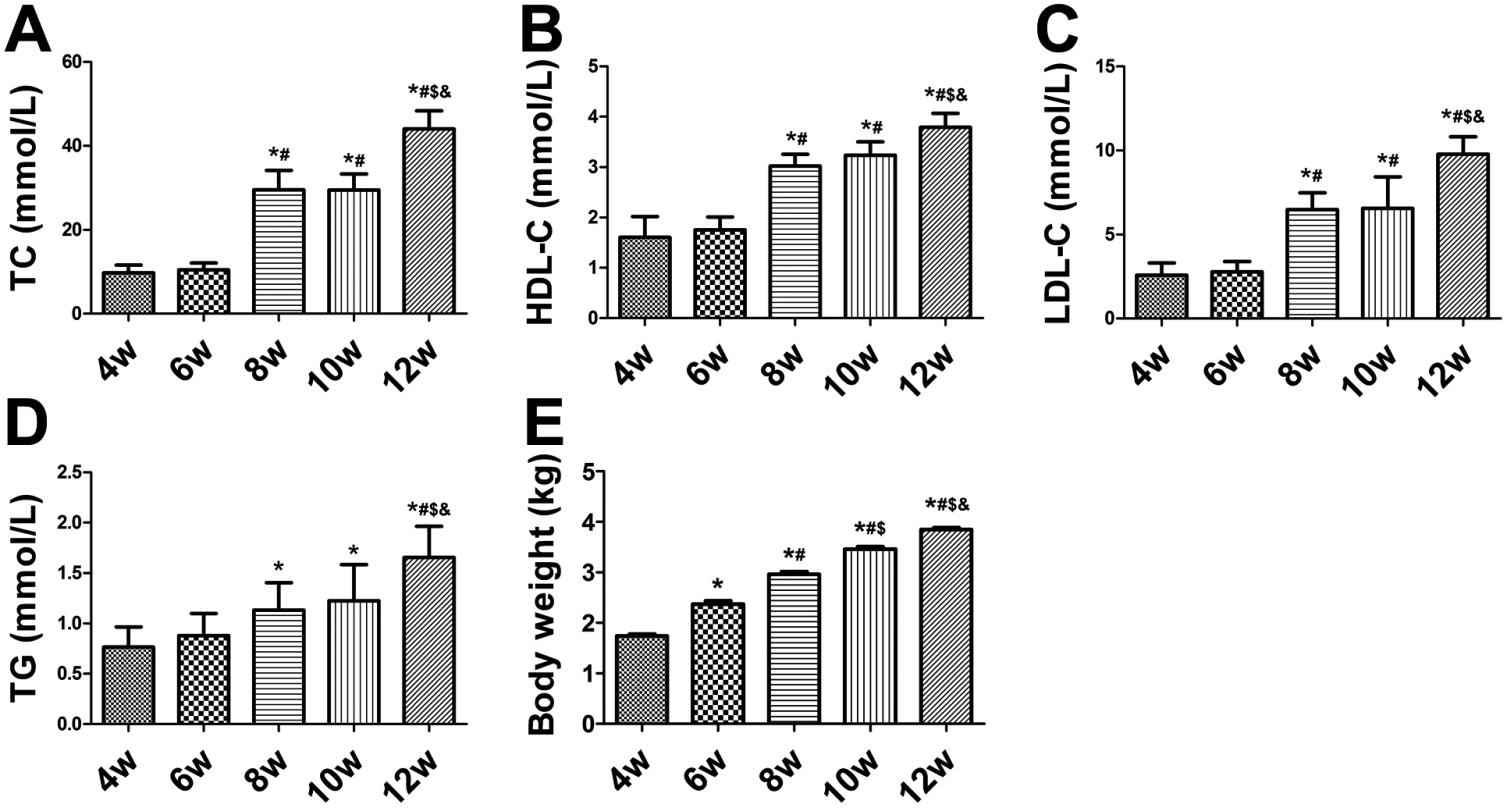
Biochemical measurements in balloon-injured rabbits. (A–D) Serum levels of total cholesterol (TC), high-density lipoprotein cholesterol (HDL-C), high-density lipoprotein cholesterol (LDL-C), and triglycerides (TG) in rabbits from weeks 4 to 12. (E) Body weights of rabbits in each group. *P<0.05 vs. week 4; ^#^ *P*<0.05 vs. week 6; ^$^ *P*<0.05 vs. week 8; ^&^ *P*<0.05 vs. week 10.

The serum levels of TC, HDL-C, LDL-C, and TG increased significantly after ingestion of the high-cholesterol diet (*P*<0.01) (Figure 1A–D). At the end of week 6, all four serum values were higher than at week 4 (*P*>0.05) (Figure 1A–D). At the end of weeks 8, 10, and 12, the serum levels of TC, HDL-C, and LDL-C were significantly higher than those at week 6 (*P*<0.05) (Figure 1A–C); however, the TG level showed no significant difference (*P*>0.05) (Figure 1D). The TG level was significantly higher at the end of week 12 than at week 6 (*P*<0.05) (Figure 1D). The body weights of the rabbits gradually increased over time (*P*<0.05) (Figure 1E).

### IVUS measurements in the balloon-injured rabbits

**Figure 2:**
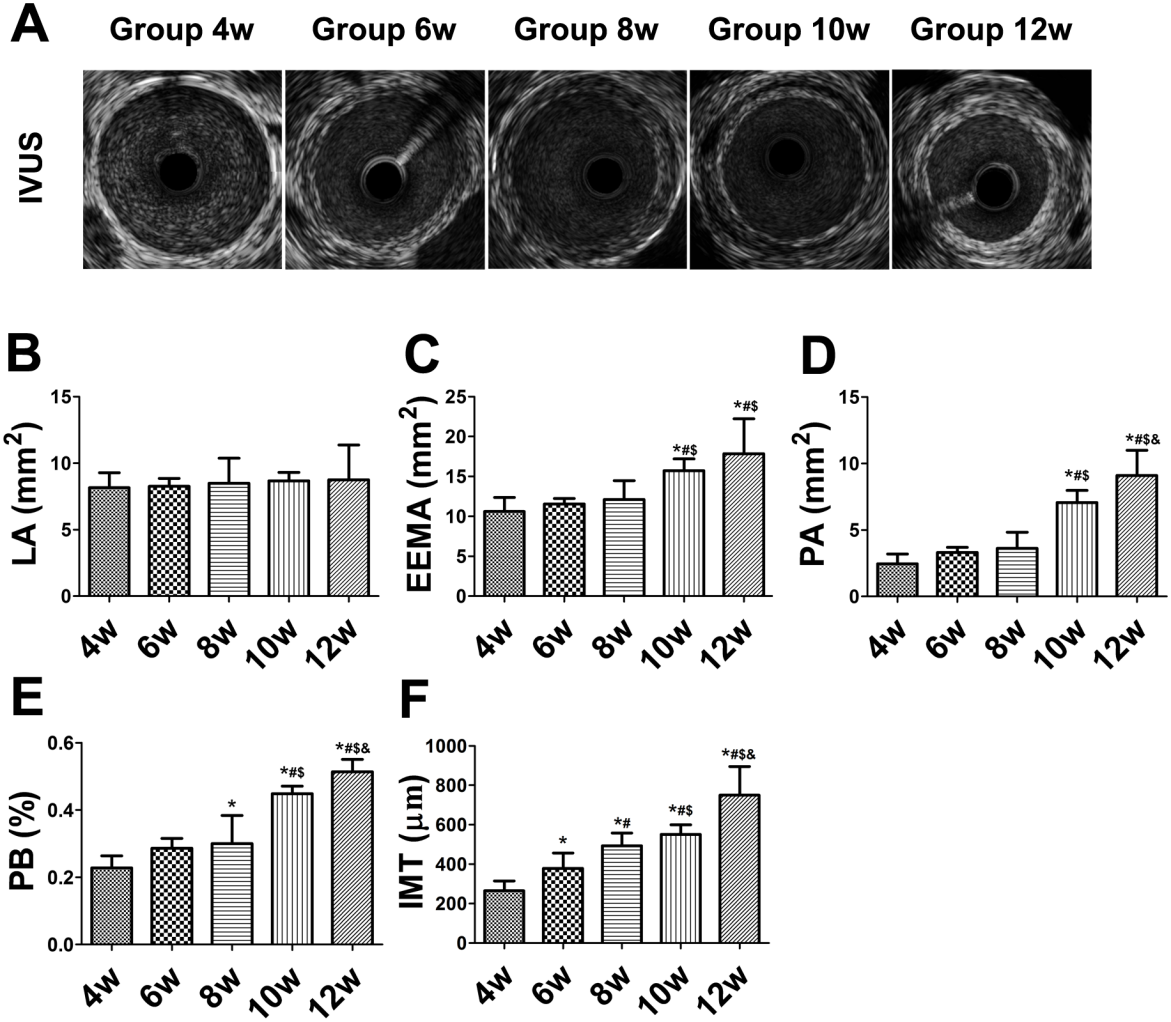
Intravascular ultrasound (IVUS) imaging and measurements in balloon-injured rabbits. (A) IVUS images. (B–F) Measurement of lumen area (LA), external elastic membrane area (EEMA), plaque area (PA), plaque burden (PB), and intima–media thickness (IMT). *P<0.05 vs. week 4; ^#^ *P*<0.05 vs. week 6; ^$^ *P*<0.05 vs. week 8; ^&^ *P*<0.05 vs. week 10.

The LA, EEMA, PA, and PB values increased throughout the duration of the experiment. The LA in the abdominal aorta did not differ among the 5 weeks (*P*>0.05) (Figure 2A, B). However, the EEMA, PA, and PB values were higher at weeks 10 and 12 than at week 4 (*P*<0.01), with no difference among weeks 4, 6, and 8 (*P*>0.05) (Figure 2A, C–E).

### Histopathological examination of the balloon-injured rabbits

The IMT of the abdominal aortic plaques increased until week 12 and was higher at week 6 than at week 4 (*P*<0.01) (Figure 2F). The IMT was significantly thicker at weeks 8, 10, and 12 than at week 6 (*P*<0.01) (Figure 2F).

### Immunohistochemical examination of the balloon-injured rabbits

**Figure 3:**
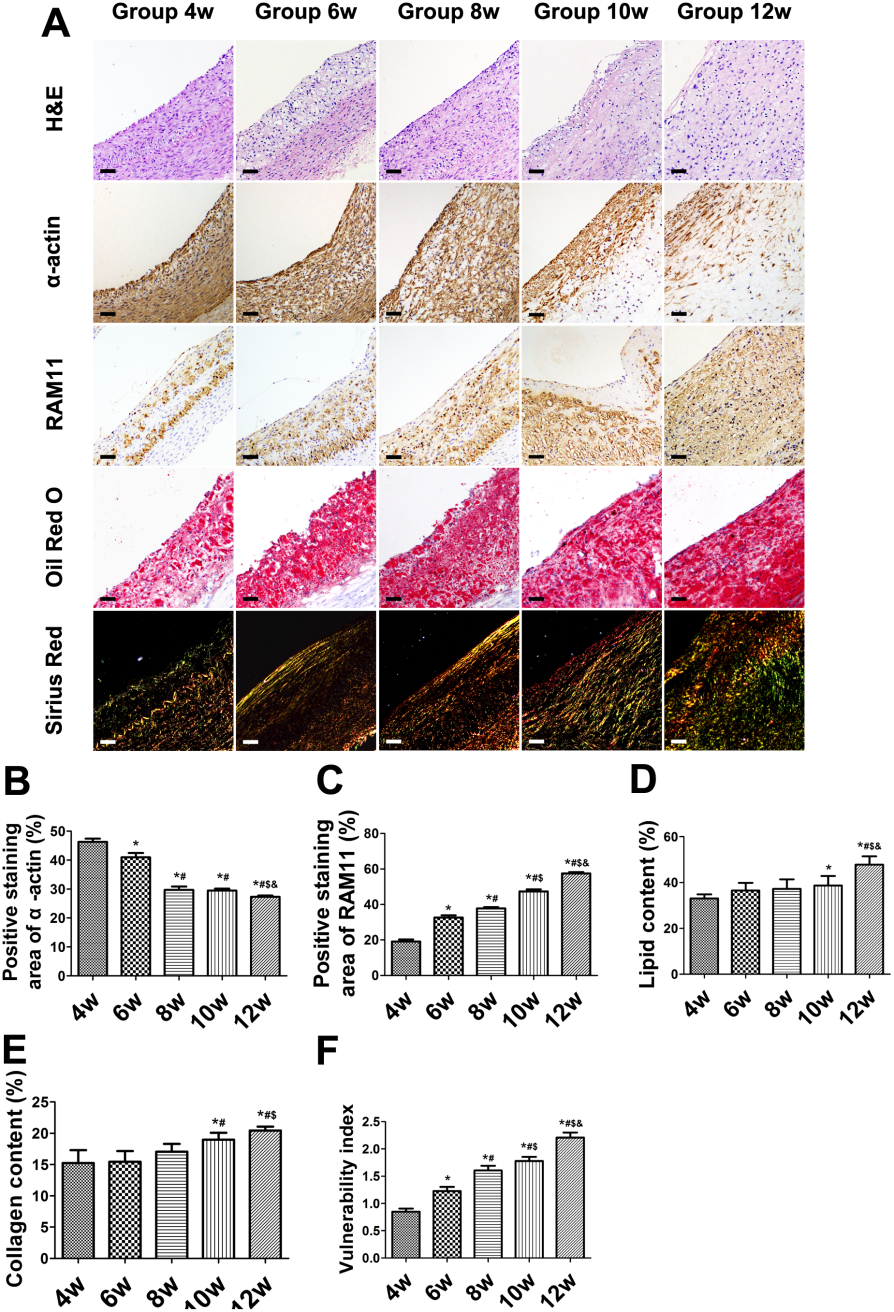
Immunohistochemical staining of plaque components in aortic plaques of balloon-injured rabbits. (A) Hematoxylin-and-eosin staining of abdominal aortic cross sections showing plaque area. α-actin staining for smooth muscle cells. RAM-11 staining for macrophages. Oil-red O staining for lipids. Sirius red staining for collagen. (B–E) Quantification of results in (A). (Bars = 20 µm). (F) Vulnerability index. *P<0.05 vs. week 4; ^#^ *P*<0.05 vs. week 6; ^$^ *P*<0.05 vs. week 8; ^&^ *P*<0.05 vs. week 10.

The areas of α-actin-positive staining in the abdominal aortic SMCs gradually decreased over time (Figure 3A, B). The areas of α-actin-positive staining within the abdominal aorta significantly decreased at all weeks with the exception of week 4 (*P*<0.01) (Figure 3A, B). The SMC plaque content was significantly lower at week 8 than 6 and at week 12 than 10 (both *P*<0.01), with no difference between weeks 8 and 10 (*P*>0.05) (Figure 3A, B). RAM-11 staining showed that the relative content of macrophages within plaques increased from weeks 4 to 12, with differences among all weeks (all *P*<0.01) (Figure 3A, C). The lipid plaque content was higher at week 12 than at all other weeks (*P*<0.01) (Figure 3A, D) with increasing plaque area. The lipid content was significantly higher at week 10 than 4 (*P*<0.05), but no significant differences were observed among weeks 4, 6, and 8 (*P*>0.05) (Figure 3A, D). The positivity of Sirius red collagen staining did not differ among weeks 4, 6, and 8. Staining was more intense at weeks 10 and 12 than at any other weeks (*P*<0.05), but no significant difference was observed between weeks 10 and 12 (*P*>0.05) (Figure 3A, E). As a result, the VI gradually increased over time, with statistically significant differences among all weeks (Figure 3F).

**Figure 4:**
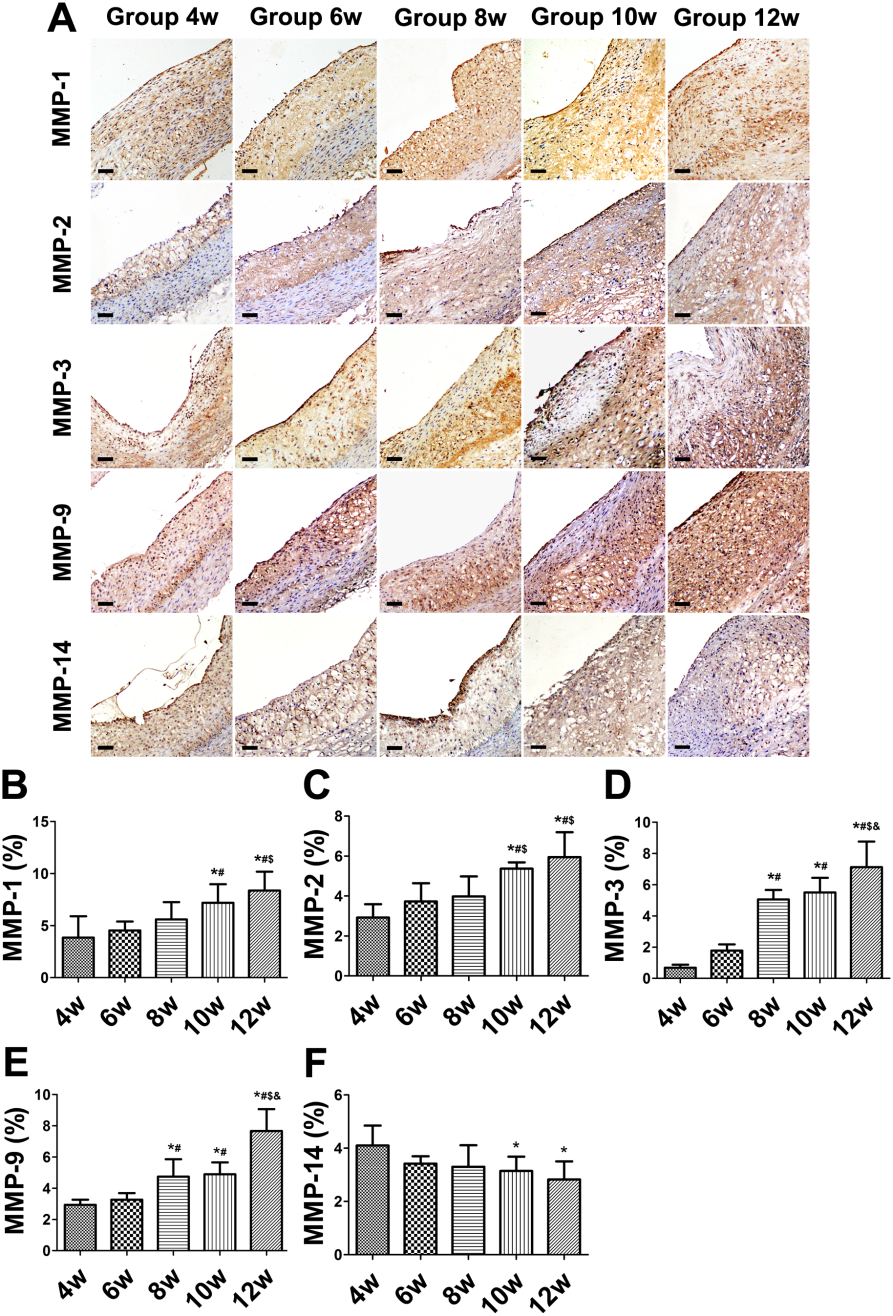
Immunohistochemical staining of matrix metalloproteinases (MMPs) and quantitative analysis in aortic plaques of balloon-injured rabbits. (A) Protein expression of MMP-1, -2,-3, -9, and -14. (B–F) Quantification. (Bars = 20 µm). *P<0.05 vs. week 4; ^#^ *P*<0.05 vs. week 6; ^$^ *P*<0.05 vs. week 8; ^&^ *P*<0.05 vs. week 10.

The expression of MMP-1, -2, -3, and -9 significantly increased from weeks 4 to 12 (Figure 4A–E). The proportion of areas showing MMP-14-positive staining substantially decreased from weeks 4 to 12 (Figure 4A, F).

**Figure 5:**
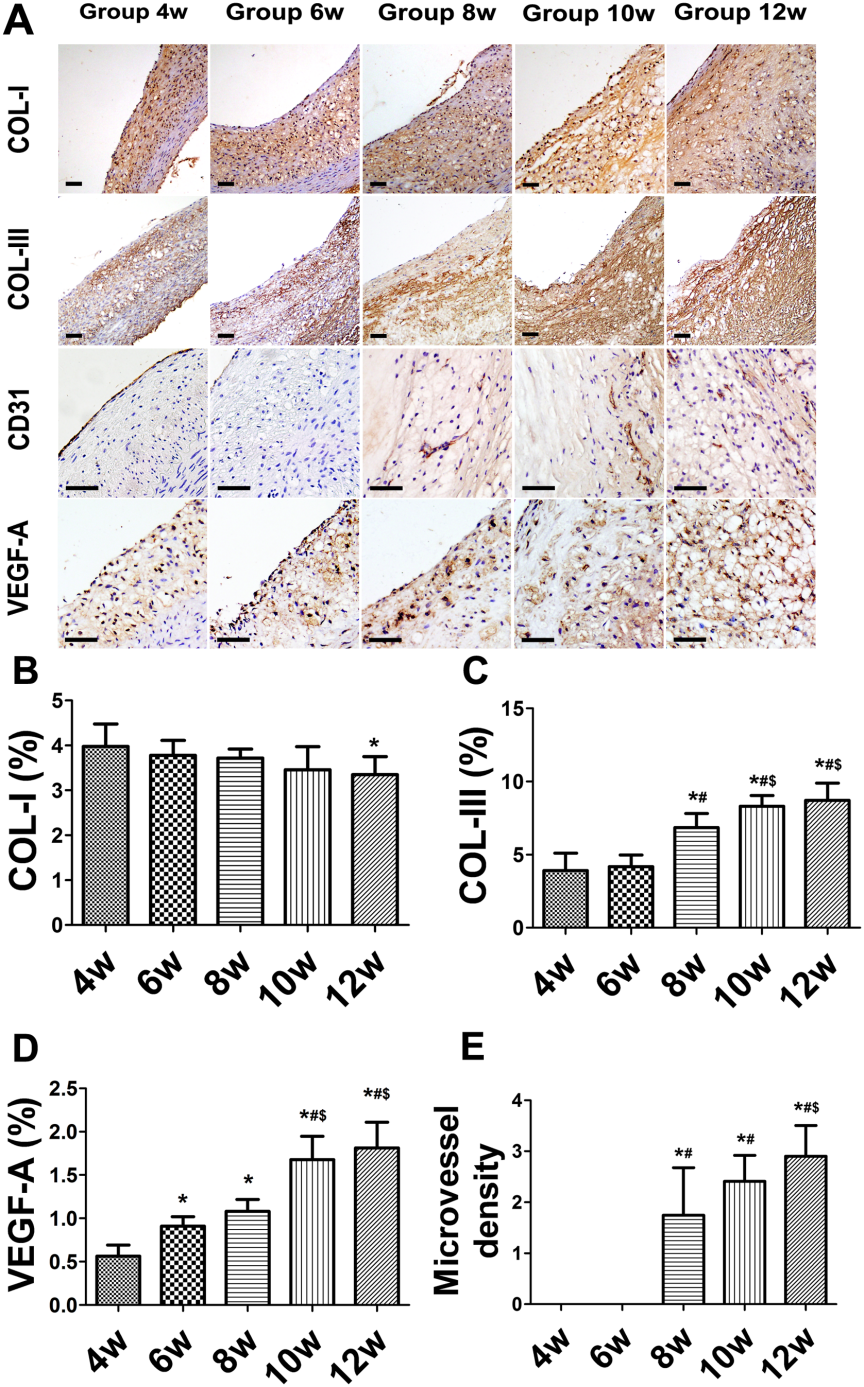
Immunohistochemical staining of collagen, CD31, and vascular endothelial growth factor A (VEGF-A) in aortic plaques of balloon-injured rabbits. (A) Protein expression of collagen I (COL-I), collagen III (COL-III), CD31, and VEGF-A. (B–E) Quantification. (Bars = 20 µm). *P<0.05 vs. week 4; ^#^ *P*<0.05 vs. week 6; ^$^ *P*<0.05 vs. week 8; ^&^ *P*<0.05 vs. week 10.

The collagen I level in plaques did not significantly differ among any weeks (*P*>0.05), but was slightly lower at week 12 than at week 4 (*P*<0.05) (Figure 5A, B). The relative content of collagen III in plaques increased from weeks 4 to 12, was higher at week 8 than at weeks 4 and 6 (*P*<0.05), and exhibited a significant change from weeks 8 to 12 (*P*<0.05) (Figure 5A, C). The expression of VEGF-A significantly increased from weeks 4 to 12 (*P*<0.05) (Figure 5A, D). Plaque neovessels appeared at week 8 (Figure 5A). The MVD was higher at week 12 than 8 (*P*<0.05), but no difference was observed between weeks 8 and 10 (both *P*>0.05) (Figure 5A, E).

### Western blot analysis in the balloon-injured rabbits

**Figure 6:**
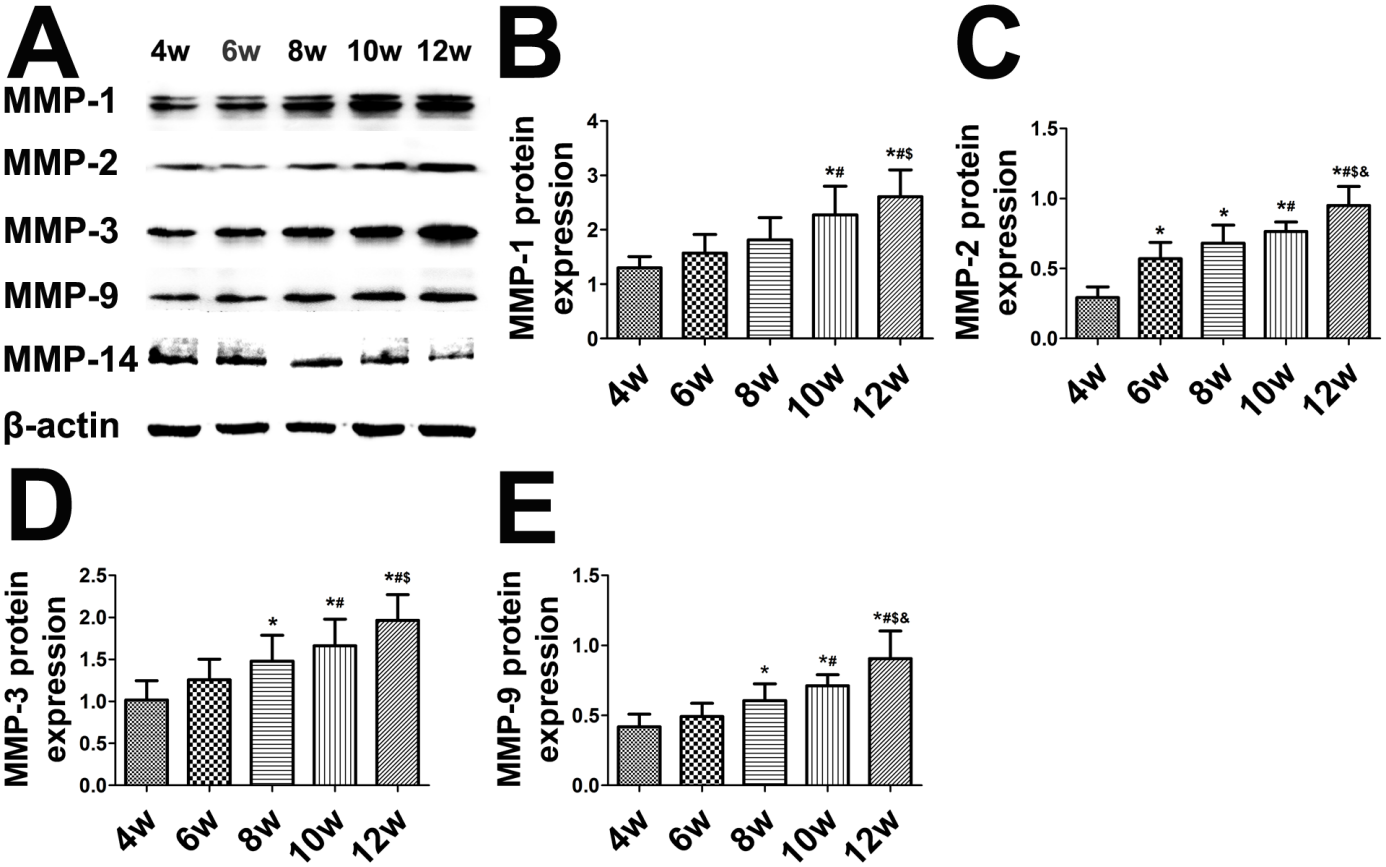
Western blot analysis and quantification of matrix metalloproteinase (MMP) protein expression in aortic plaques of balloon-injured rabbits. (A) Protein expression of MMP-1, -2, -3, -9, and -14. (B–F) Quantification. *P<0.05 vs. week 4; ^#^ *P*<0.05 vs. week 6; ^$^ *P*<0.05 vs. week 8; ^&^ *P*<0.05 vs. week 10.

The expression of MMP proteins exhibited the same trends as shown in the immunohistochemical results. The expression of MMP-1, -2, -3, and -9 significantly increased from weeks 4 to 12 (Figure 6A–E), while that of MMP-14 substantially decreased from weeks 4 to 12 (Figure 6A, F).

### Correlation analysis in the balloon-injured rabbits

The correlation analysis results in the balloon-injured animals are shown in Table 1. All correlations between the VI and MMP-1, -2, -3, and -9 were positive (r = 0.767, 0.809, 0.890, and 0.887, respectively). The expression of MMP-1, -2, -3, and -9 was positively correlated with both the MVD in plaque (r = 0.762, 0.813, 0.884, and 0.769, respectively) and the VEGF-A in plaque (r = 0.760, 0.762, 0.858, and 0.789, respectively). The correlations between MMP-14 and the VI, MVD, and VEGF-A were all negative (r = –0.556, –0.424, and –0.525, respectively). The correlation between the VI and MVD was positive (r = 0.846).

**Table 1:** Spearman correlations between matrix metalloproteinase (MMP) levels and the vulnerability index (VI), microvascular density (MVD), and vascular endothelial growth factor A (VEGF-A) level in balloon-injured rabbits.

	VI	MMP-1	MMP-2	MMP-3	MMP-9	MMP-14
**VI**	1.000	0.767**	0.809**	0.890**	0.887**	–0.556*
**MVD**	0.846**	0.762**	0.813**	0.884**	0.769**	–0.424*
**VEGF-A**	0.898**	0.760**	0.762**	0.858**	0.789**	–0.525**

*Statistically significant, *P*<0.05.

**Statistically significant, *P*<0.01.

## Discussion

The most important finding in this study is that in this rabbit model of atherosclerosis, MMP-1, -2, -3, and -9 were positively correlated and MMP-14 was negatively correlated with intraplaque angiogenesis at the advanced stages of atherosclerosis.

Recent studies have found that MMPs participate and are indispensible in the process of angiogenesis. MMP-1 deficiency significantly decreases angiogenesis via the protease-activated receptor-1 pathway in lung tumors [8]. MMP-1 and -3 can degrade perlecan in basement membranes, releasing basic fibroblast growth factor (basic FGF) [9]. Likewise, MMP-2 and -3 degrade the ECM proteoglycan decorin, releasing latent tissue growth factor 1. MMP-2 and -9 can cleave latency-associated peptide to activate tissue growth factor β1, MMP-2 and MMP-9 have been shown to be critical for the “angiogenic switch in tumor angiogenesis [10], [11]. However, different studies have shown different results. MMP-2 reportedly cleaves the ectodomain of FGF receptor 1, which retains FGF-binding activity, but is unable to signal and thus modulate the biological availability and mitogenic and angiogenic activities of FGFs [15]. In another study, reductions in MMP-9 levels by pharmacological methods in either wild-type or α1-knockout mice resulted in reduced angiostatin levels and increased tumor growth and vascularization [13]. Stable overexpression of MMP-9 in a mouse colon carcinoma cell line resulted in increased angiostatin levels and decreased tumor growth and angiogenesis *in vivo* [13]. The present study showed that MMP-1, -2, -3, and -9 are all strongly positive correlated with the MVD. Therefore upregulation of MMP-1, -2, -3, and -9 may enhance intraplaque angiogenesis at the advanced stage of atherosclerosis in rabbits.

A previous study showed that selective inhibition of MMP-14 blocks tumor angiogenesis [12]. Another showed that MMP-14 generates endogenous angiogenesis inhibitors by proteolytic cleavage of plasma proteins and ECM components. MMP-14 cleaves endoglin, a transforming growth factor-β coreceptor, at a site located close to the transmembrane domain. MMP-14 also upregulates the level of soluble endoglin, thus reducing the occurrence of spontaneous and VEGF-induced endothelial sprouting and inhibiting angiogenesis within tumors [14]. A placental study showed that MMP-14 acts as the cleavage protease for endoglin [23]. Cleavage of collagen XVIII by MMP-14 can generate endostatin, an angiogenesis inhibitor that blocks VEGF-induced endothelial cell migration [24]. In the present study, we found that MMP-14 is negatively correlated with angiogenesis at the advanced stages of atherosclerosis in rabbits. Therefore, downregulation of MMP-14 may participate in intraplaque angiogenesis at the advanced stages of atherosclerosis.

Angiogenesis is a critical factor in the development and progression of atherosclerosis. Pathological examination of unstable lesions has demonstrated that plaque rupture is associated with an increased density of microvessels. Angiogenesis of the intima is a consistent feature of plaque development in atherosclerosis [25]. Previous studies showed that the number of vasa vasorum was two-fold higher in vulnerable plaques and up to four-fold higher in ruptured plaques than in stable plaques with severe luminal narrowing [26], [27]. Mofidi *et al.* found strong associations between angiogenesis in atherosclerotic carotid plaques and plaque vulnerability [28]. The present study also showed that angiogenesis is correlated with plaque instability.

MMPs are correlated with changes associated with plaque vulnerability, such as macrophage ingress and apoptosis as well as loss of collagen and elastin [29], [30]. MMP-3-knockout decreased the incidence of elastin breaks [31], implying greater stability. Luttun et al. showed that MMP-9-knockout reduced plaque size, macrophage content, and elastin breaks, leading to plaque stability [32]. Overexpression of an autoactivated form of MMP-9 resulted in substantially greater plaque instability [33]. The correlation analysis in our study revealed that MMP-1, -2, -3, and -9 were positively correlated with the VI in plaques and that MMP-14 was significantly negatively correlated with the VI.

In the present study, the levels of MMP-1, -2, -3, and -9 were positively correlated with the expression of VEGF-A within plaques. MMPs are reportedly able to enhance the bioactivity of growth factors, and their expression is induced by angiogenic factors *in vitro*. Degradation of ECM releases membrane-sequestered VEGF [34]. MMP-1 promotes the expression of VEGF receptor 2 via stimulation of protease-activated receptor-1 and activation of NF-κB in endothelial cells [35]. VEGF-A-dependent phosphorylation of intracellular signaling molecules such as extracellular signal-regulated kinase and Akt has been observed within endothelial cells [35]. Connective tissue growth factor forms an inactive complex with VEGF-A, and cleavage of connective tissue growth factor by MMP-1 or -3 releases active VEGF-A within endothelial cells [36]. MMP-3 activates several MMPs, including proMMP-1, -7, and -9. Activated MMP-7 can in turn activate proMMP-1 and proMMP-9 [37], [38]. Suppression of MMP-2 decreases integrin-αvβ3-mediated induction of PI3K/AKT, thus leading to decreased VEGF-A expression in lung cancer cells [39]. Recent studies have also demonstrated that adenovirus-mediated transfer of siRNA against MMP-2 results in impaired expression of VEGF and tumor-induced angiogenesis both *in vitro* and *in vivo* [40]. MMP-9 releases VEGF-A bound to proteoglycans within the ECM [41], thus enhancing the bioavailability of VEGF-A and potentially influencing plaque angiogenesis. Overexpression of MMP-9 in human breast cancer cells increases VEGF–VEGF receptor 2 complex formation and tumor angiogenesis [42]. Thus, MMP-1, -2, -3, and -9 can promote the expression of VEGF-A either *in vitro* or within tumors. The processes triggered positive feedback cycles, enhancing the development of angiogenesis and finally leading to intraplaque hemorrhage and plaque rupture. These findings strongly support the influence of widespread MMP-1, -2, -3, and -9 expression within plaques on angiogenesis, in part by the role of these MMPs in activating VEGF-A.

Another important finding of the present study is that plaque neovessels first appeared at week 8. Angiogenesis is one of the key therapeutic factors in stabilization of atherosclerotic plaques; thus, inhibition of angiogenesis seems to be particularly important. Interest in intraplaque angiogenesis has been spurred by the potential to target plaque neovascularization with angiogenesis inhibitors [43]. Identification of the optimal time point at which to inhibit angiogenic growth within atherosclerotic lesions may lead to the development of therapies designed to stabilize plaques. Our findings may lead to the identification of this time point, thus assisting in the development of targeted drug or gene intervention therapy for intraplaque angiogenesis.

The present study contains several limitations. First, the sample size was relatively small. Further studies with larger samples are required to confirm our primary conclusions. Second, although the detailed molecular mechanisms of the influence of MMPs on plaque angiogenesis were investigated, further *in vitro* studies are required to fully elucidate the signal transduction pathways involved. Third, MMP gene interference is a preferred approach with which to determine the specific correlation between MMPs and intraplaque angiogenesis. Finally, the plaque formation in our animal model may not entirely simulate that in patients with acute coronary syndrome; the plaque-stabilizing effect of MMPs requires evaluation in clinical trials.

In conclusion, as shown in this rabbit model of atherosclerosis, upregulation of MMP-1, -2, -3, and -9 and downregulation of MMP-14 may participate in intraplaque angiogenesis at the advanced stages of atherosclerosis. Further investigation of MMPs may provide a novel approach for the prediction and treatment of vulnerable atherosclerotic plaques.

## Supporting Information

Figure S1 **Biochemical measurements of rabbits in the control group.** The serum levels of total cholesterol (TC), high-density lipoprotein cholesterol (HDL-C), high-density lipoprotein cholesterol (LDL-C), and triglycerides (TG) significantly increased after ingestion of an atherogenic diet for 12 weeks (*P*<0.05). The body weights of the rabbits gradually increased over time (*P*<0.05).
(TIF)

Figure S2 **Intravascular ultrasound (IVUS) imaging of rabbits in the control group.** Only scarce plaque was present within the abdominal aorta after ingestion of an atherogenic diet for 12 weeks in the control group.
(TIF)

Figure S3 **Hematoxylin-and-eosin (H&E) staining of abdominal aorta of rabbits in the control group.** Only fatty streaks with lipid infiltration and no intimal injury were present in the abdominal aorta among rabbits without intimal injury after ingestion of an atherogenic diet for 12 weeks (bars = 20 µm).
(TIF)

Figure S4 **Immunohistochemical staining of matrix metalloproteinases (MMPs) and CD31 in the abdominal aorta of rabbits in the control group.** Rare MMPs are stained within smooth muscle cells in the control group. CD31 staining showed no angiogenesis after ingestion of an atherogenic diet for 12 weeks (bars = 20 µm).
(TIF)
