# Supplementary figures and images for: Specific Matrix Metalloproteinases Play Different Roles in Intraplaque Angiogenesis and Plaque Instability in Rabbits
Source: PLoS One. 2014 Sep 18;9(9):e107851. doi: 10.1371/journal.pone.0107851 (PMC4169444; doi:10.1371/journal.pone.0107851)

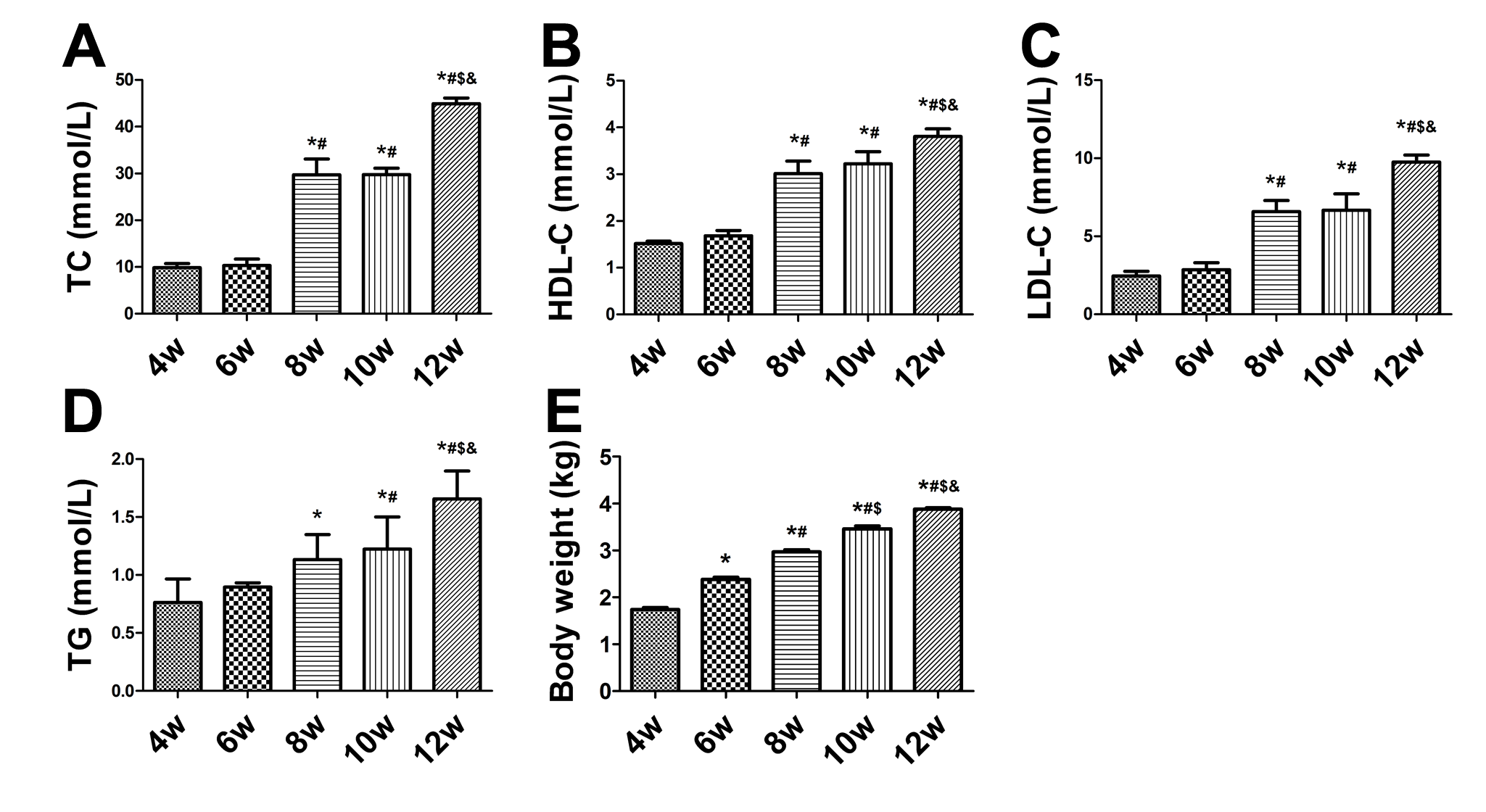

Supplement: Figure S1 — Biochemical measurements of rabbits in the control group. The serum levels of total cholesterol (TC), high-density lipoprotein cholesterol (HDL-C), high-density lipoprotein cholesterol (LDL-C), and triglycerides (TG) significantly increased after ingestion of an atherogenic diet for 12 weeks (P<0.05). The body weights of the rabbits gradually increased over time (P<0.05). (TIF) [file pone.0107851.s001.tif]

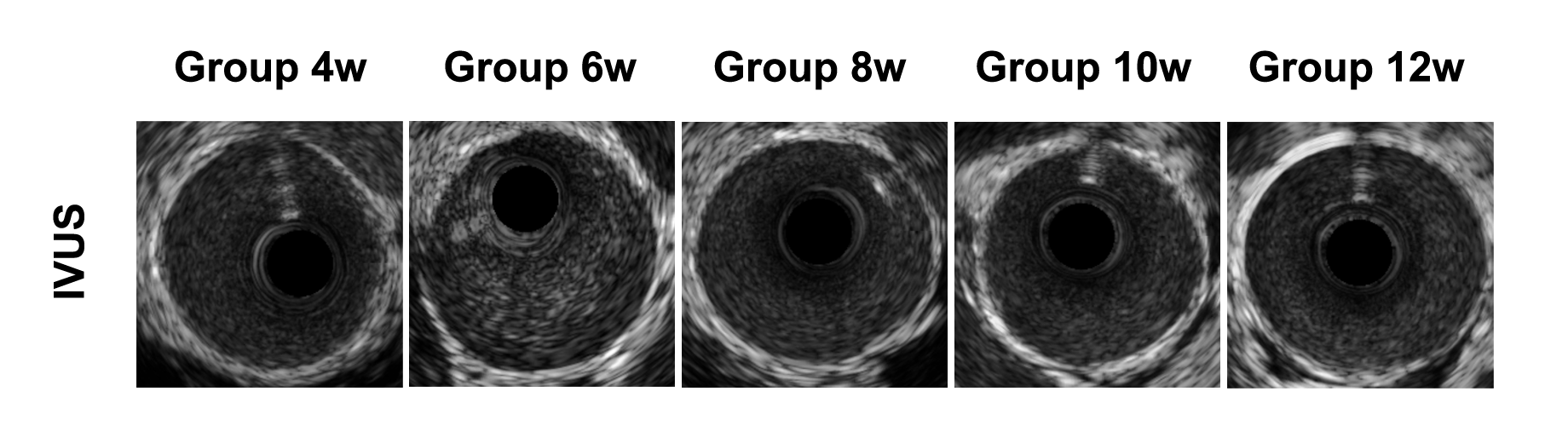

Supplement: Figure S2 — Intravascular ultrasound (IVUS) imaging of rabbits in the control group. Only scarce plaque was present within the abdominal aorta after ingestion of an atherogenic diet for 12 weeks in the control group. (TIF) [file pone.0107851.s002.tif]

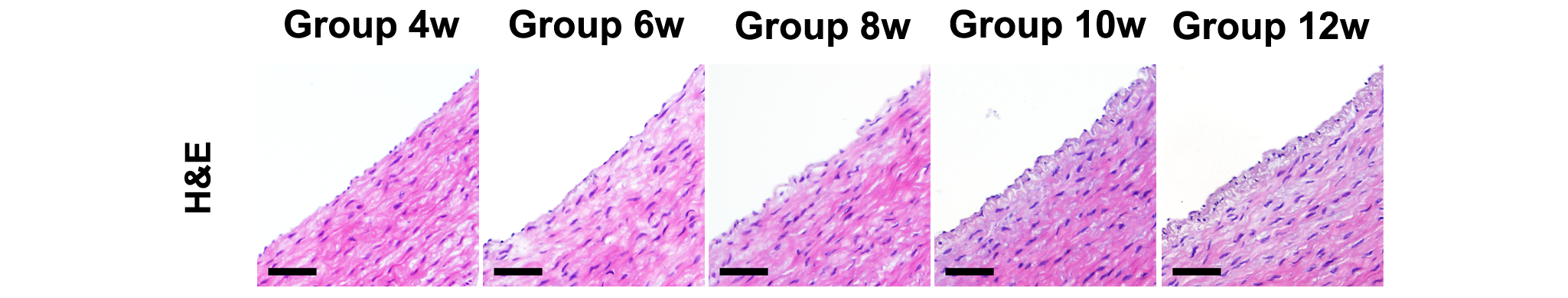

Supplement: Figure S3 — Hematoxylin-and-eosin (H&E) staining of abdominal aorta of rabbits in the control group. Only fatty streaks with lipid infiltration and no intimal injury were present in the abdominal aorta among rabbits without intimal injury after ingestion of an atherogenic diet for 12 weeks (bars = 20 µm). (TIF) [file pone.0107851.s003.tif]

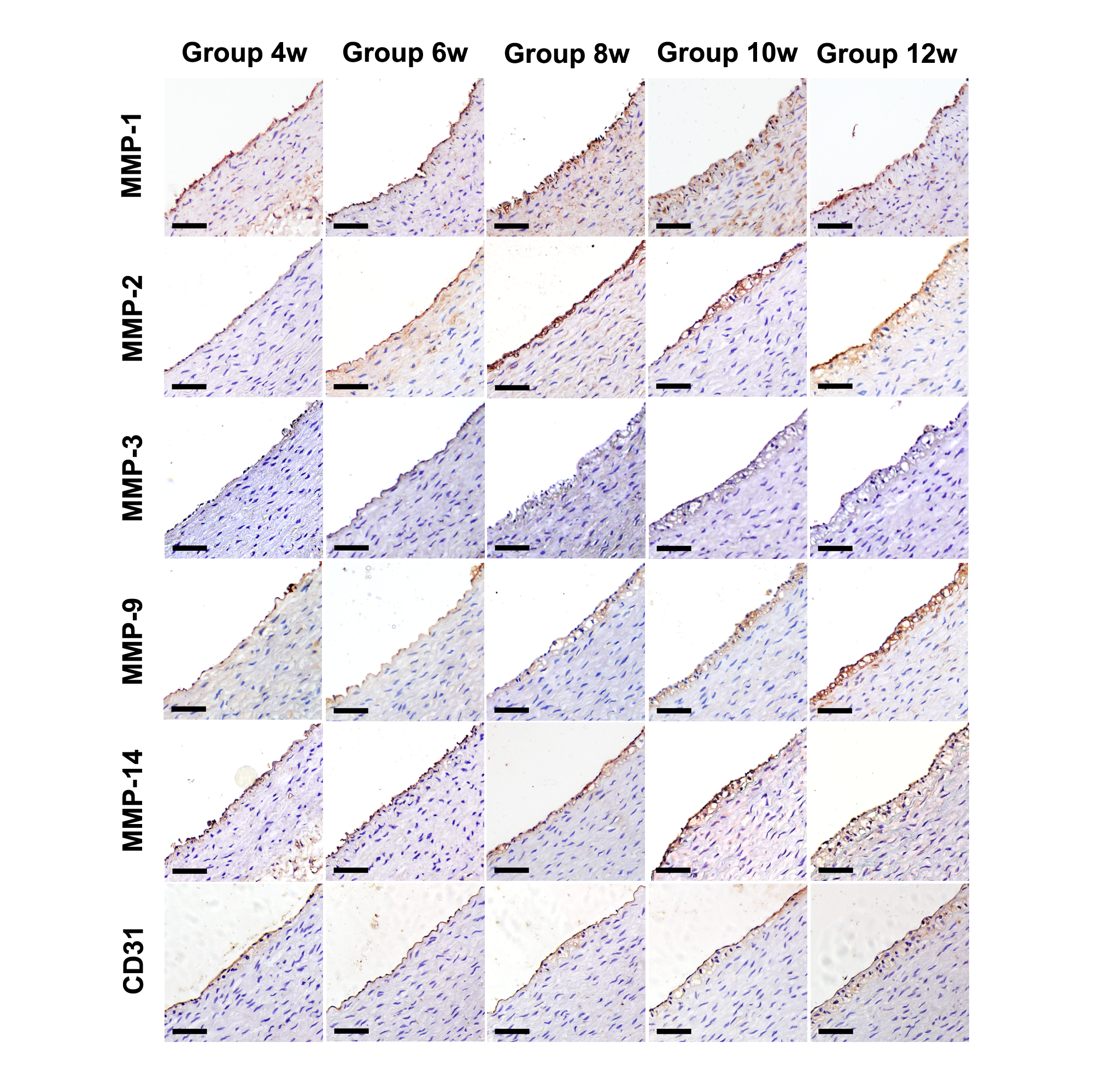

Supplement: Figure S4 — Immunohistochemical staining of matrix metalloproteinases (MMPs) and CD31 in the abdominal aorta of rabbits in the control group. Rare MMPs are stained within smooth muscle cells in the control group. CD31 staining showed no angiogenesis after ingestion of an atherogenic diet for 12 weeks (bars = 20 µm). (TIF) [file pone.0107851.s004.tif]
